# High-Throughput Flow Cytometry Combined with Genetic Analysis Brings New Insights into the Understanding of Chromatin Regulation of Cellular Quiescence

**DOI:** 10.3390/ijms21239022

**Published:** 2020-11-27

**Authors:** Yasaman Zahedi, Mickael Durand-Dubief, Karl Ekwall

**Affiliations:** Department of Biosciences and Nutrition, Neo Building, Karolinska Institute, SE-141 83 Huddinge, Sweden; yasaman.zahedi@ki.se (Y.Z.); mickael.duranddubief@gmail.com (M.D.-D.)

**Keywords:** cellular quiescence, G_0_, fission yeast, chromatin, SAGA, Rpd3, Ino80, DNA repair

## Abstract

Cellular quiescence is a reversible differentiation state when cells are changing the gene expression program to reduce metabolic functions and adapt to a new cellular environment. When fission yeast cells are deprived of nitrogen in the absence of any mating partner, cells can reversibly arrest in a differentiated G_0_-like cellular state, called quiescence. This change is accompanied by a marked alteration of nuclear organization and a global reduction of transcription. Using high-throughput flow cytometry combined with genetic analysis, we describe the results of a comprehensive screen for genes encoding chromatin components and regulators that are required for the entry and the maintenance of cellular quiescence. We show that the histone acetylase and deacetylase complexes, SAGA and Rpd3, have key roles both for G_0_ entry and survival during quiescence. We reveal a novel function for the Ino80 nucleosome remodeling complex in cellular quiescence. Finally, we demonstrate that components of the MRN complex, Rad3, the nonhomologous end-joining, and nucleotide excision DNA repair pathways are essential for viability in G_0._

## 1. Introduction

Cellular quiescence is a reversible differentiation state when cells are changing the gene expression program to reduce metabolic functions and adapt to a new cellular environment. The ability to exit from the cell cycle into quiescence (G_0_) is an important longevity strategy for unicellular organisms to ensure cell survival during times of limited nutrients. In recent years, the regulation of entry into quiescence has been shown to involve complex relationships between different molecular mechanisms.

Here we systematically investigate the chromatin regulators required for entry and survival during quiescence in fission yeast (*Schizosaccharomyces pombe*) as a genetic model organism. Fission yeast has been shown to be an excellent model to study cellular quiescence since switching between vegetative and quiescent phases can easily be regulated by nitrogen availability in the media [1,2]. When fission yeast cells are starved for nitrogen source, they rapidly divide twice without growth and arrest in the G_1_ stage of the cell cycle and then enter into cellular quiescence (G_0_) to form small and round cells with condensed nuclei. During this process, the physiology and the transcription profile of quiescent *S. pombe* cells are considerably reprogrammed to adapt to the physiological challenge [3,4,5]. Previous studies have used genetic screens in *S. pombe* to investigate key genes involved in the entry into and exit from quiescence and have identified a broad range of cellular processes such as stress-response cell cycle signaling pathways, endosome formation, RNA transcription, RNA processing, protein translation, lipid biosynthesis, cell-wall morphogenesis, membrane function, autophagy, RNA interference, and heterochromatin assembly [3,4,6,7,8]. We recently found a role for the RNA polymerase II-associated Paf1 complex controlling heterochromatin dynamics being essential for survival during quiescence [9].

In this work, we aim to systematically investigate specific genes and pathways in chromatin regulation, such as transcription, chromatin remodeling, and DNA repair pathways involved in the entry and the maintenance of the quiescence stage using high-throughput flow cytometry. Flow cytometry has many advantages to measure accurately properties of cells, such as the size, metabolic activities, or DNA content [10]. Flow cytometry is an excellent method for detailed cell cycle analysis using fission yeast cells [11]. Recent technological advances allow high-throughput flow cytometry analysis using plate format offering the possibility to investigate multiple phenotypes in large mutant collections. Thus, systematic high-throughput flow cytometry analysis on mutant libraries provides a host of biological insights pertinent to quiescence compared to traditional approaches. Here we analyze the cell cycle behavior and cell mortality of each haploid deletion mutant in a comprehensive custom-made gene deletion library focused on chromatin regulation. The Rpd3 histone deacetylase complex has previously been shown to play an important role in repression of transcription during quiescence in budding yeast [12]. We show that Rpd3 is essential for G_0_ also in fission yeast and we find an additional role for the SAGA histone acetylation complex in this model organism. We reveal a novel function for the Ino80 nucleosome remodeling complex during cellular quiescence. Finally, we show that several DNA repair pathways are essential for survival in G_0_.

## 2. Results

### 2.1. Rationale of the Experimental Approach

We used a collection of 3418 haploid fission yeast auxotrophic strains, each harboring a unique gene deletion of a nonessential gene [13]. Since quiescence is induced by nitrogen starvation in fission yeast and this process requires prototrophic strains, we crossed the strain collection with a prototrophic wild-type strain with nonswitchable mating type (*h-smt-0*) and selected prototrophic *h-*progeny by linkage to the *leu1 +* locus as was previously described [6]. The resulting gene deletion library can respond to nitrogen starvation by entry into quiescence. In order to study the role of genes involved in chromatin regulation and transcription in quiescence using high-throughput cytometry, a smaller custom-made library was constructed. We used gene ontology (GO) annotations available at https://www.pombase.org to select strains from the large collection that carry gene deletions involved in chromatin, DNA binding, or transcription processes. This resulted in a custom-made library containing 740 mutant strains (see Materials and Methods).

**Figure 1:**
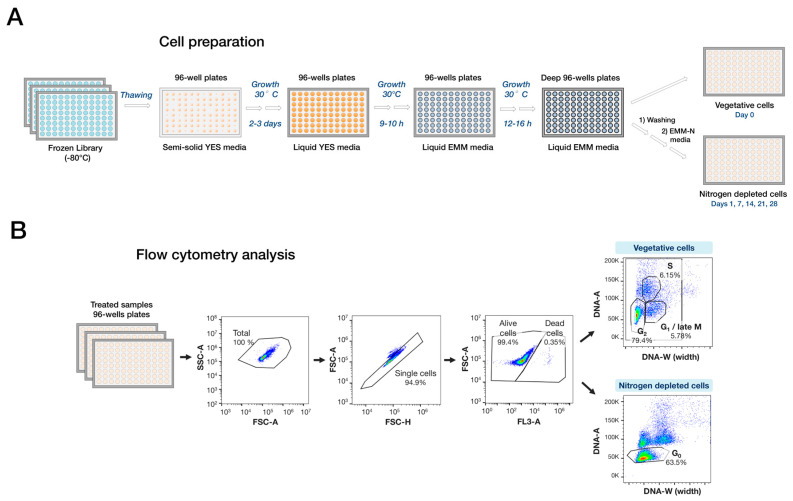
Workflow of high-throughput cell preparation and flow cytometry analysis. (**A**) Preparation of the library for high-throughput flow cytometry analysis. Cells were harvested at different time points before and after nitrogen depletion in the EMM-N media. (**B**) Overview of the gating strategy for flow cytometry analysis. After sample collection at different time points, cells were prepared both for viability and DNA content measurements by double staining. Doublet signals were excluded through FSC-A/SSC-A filtering and single cells were analyzed for mortality. The viable cells (FL3A:660A-) were then measured for DNA content and the populations of G_2_ cells and G_0_ cells where determined. During nitrogen depletion, G_0_ cells (DNA-A low/DNA-W low; gate marked in the right panel) were separated from G_2_ (DNA-A high/DNA-W low) and G1/M cells (DNA-A high/DNA-W high).

To perform flow cytometry using high-throughput, each strain in the custom deletion library was grown in duplicate spots at 30 °C on rich media (YES) 96-well plates, transferred to liquid YES followed by growth in EMM (Edinburgh Minimal Media) at 30 °C, followed by washes and resuspension in EMM-N medium lacking nitrogen (Figure 1A and Figure S1). Cell samples were harvested at the proliferative stage (day 0) and after nitrogen depletion (day 1, 7, 14, 21, and 28) and subjected to multiplex flow cytometry analysis.

For flow cytometry, a specific gating strategy was used in order to monitor the phenotype of each deletion mutant (Figure 1B and Figure S2). Briefly, signals from doublet cells were excluded using a specific gating method using the FSC-A and FSC-H channels and the signals from single cells were then analyzed to determine the fraction of dead cells (% mortality) in each mutant. The FL3A filter was used to distinguish alive and dead cells using the R660 channel (see Section 4). Next, since we also wanted to assess defects in the cell cycle behavior for each strain, the signals were analyzed for the proportion of cells in G_2_ or G_0_ phases using the DNA-A and DNA-W channels of the flow cytometer [11]. G_0_ cells (DNA-A low/DNA-W low) were separated from other cell populations (Figure 1B).

**Figure 2:**
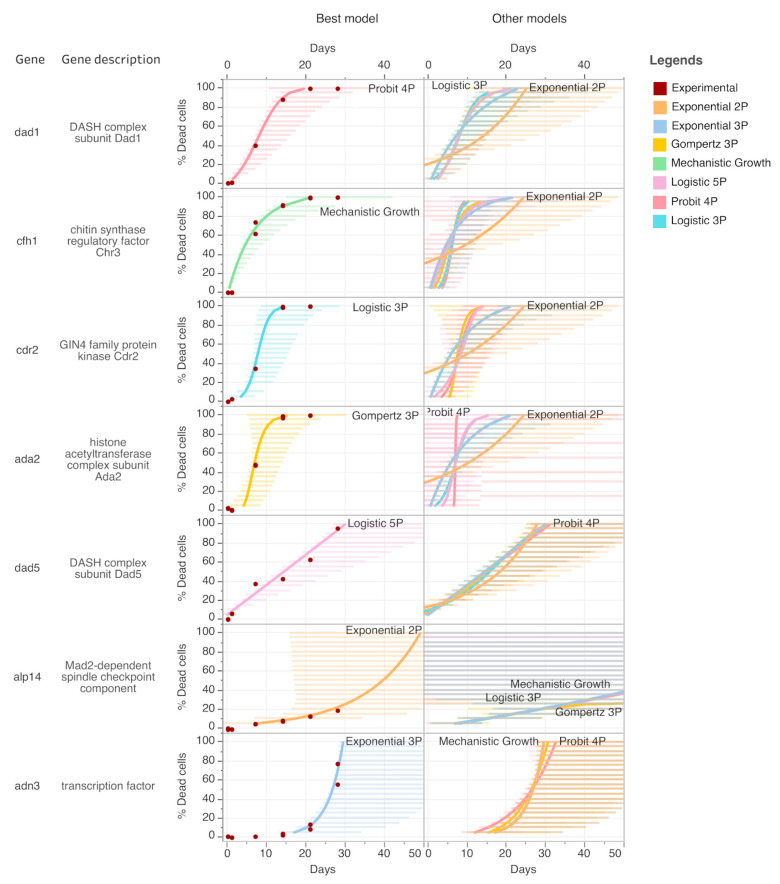
Prediction of mortality during quiescence using nonlinear models. Examples of data showing the selection procedure used for the optimal nonlinear model (Logistic 3P, Mechanistic Growth, Gompertz 3P, Logistic 5P, Exponential 3P, Exponential 2P, or Probit 4P) (see Section 4 and Figure S3). The left panel shows the experimental data of mortality measured at different time points in the nitrogen-depleted cultures and the curve fit of the optimal nonlinear model that was applied. The right panel shows the curve fit of the other tested nonlinear models.

**Figure 3:**
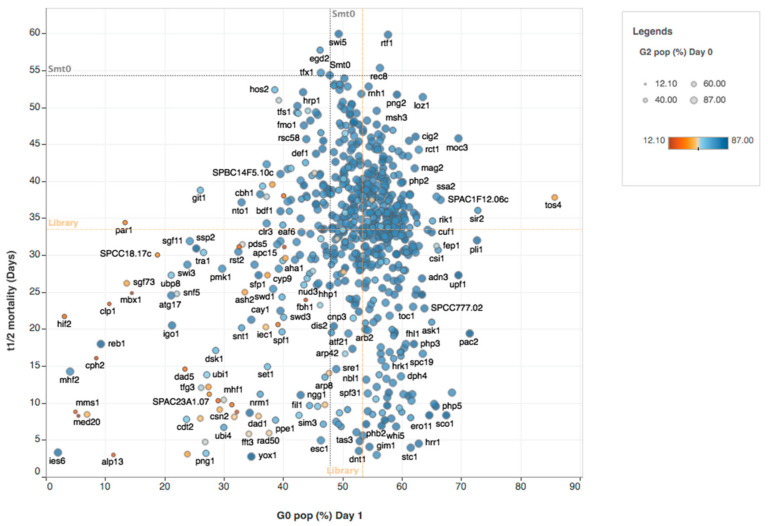
Multiphenotypic representation of cell cycle stages and predicted half-time mortality before and during quiescence by nitrogen starvation. Predicted half-time mortality (days) is represented on the Y-axis and the percentage of G_0_ cells is shown on the X-axis. The color of each dot shows the percentage of G_2_ cells at time 0 before nitrogen starvation and the dot sizes indicate the percentage of G_2_ cells after one day of starvation. Orange dashed lines indicate the respective median values of all mutants analyzed for each axis. Grey dashed lines indicate the value for *smt-0* wild-type strain for each axis.

We noticed that when cell mortality was measured over time by high-throughput flow cytometry before and after nitrogen depletion in the media, the profile of cell mortality during quiescence could be drastically different between the mutants following different trends. To distinguish accurately between these different quiescence phenotypes, we tested seven nonlinear models to determine the optimal rate of cell mortality for each mutant individually (see Figure 2 and Section 4). According to these criteria, the time for 50% (T_1/2_) and the time for 25% (T_1/4_) mortality were determined for each mutant (Figure 3 and Table S1). Most of the mutants were found to follow exponential 2P or exponential 3P models of cell mortality (Figure S3).

The extrapolated results using the nonlinear models are depicted together with the percentage of G_2_ cells at time 0 and the percentage of G_0_ cells after one day of starvation as a multiphenotypic representation for 684 haploid mutants of the library (Figure 3 and Table S1). It was clear that most of the analyzed strains showed similar half-time mortality curves compared to the control strain (*smt-0* wild-type) with values for T_1/2_ > on average about 35 days for the entire library. However, for a substantial fraction of the library strains, high mortality phenotypes could be observed before or after the transition to G_0_.

### 2.2. Hierarchical Clustering of Mutant Phenotype Patterns in Quiescence

**Figure 4:**
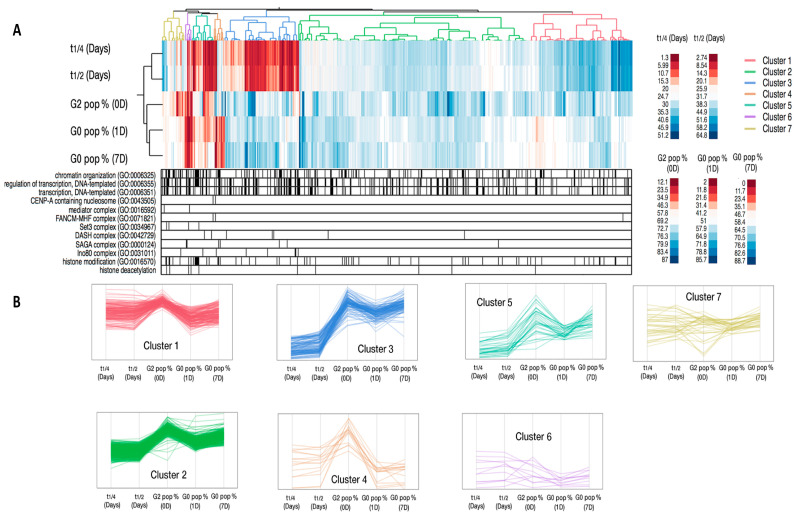
Hierarchical clustering of mutant phenotype patterns. (**A**) Heat map showing the seven phenotypic clusters. T_1/2_ (Days) and T_1/4_ (Days) indicate the extrapolated time in days for 50% or 25% cell mortality, respectively. The G_2_ percentage was measured at time 0. The G_0_ percentages after 1 day and 7 days of nitrogen starvation are indicated. The seven identified clusters are shown in different colors in the dendogram (top). The black lines under the heat map show relevant gene ontology (GO) processes for each gene. (**B**) Normalized intensities of the phenotypic data illustrate the characteristics of each gene cluster (each line represents one mutant strain). The seven different identified clusters are shown in the same colors as in panel A.

Next, we used the following five different phenotype measurements for clustering of the mutant data: extrapolated 50% and 25% of cell mortality time points, the G_2_% at time 0, the G_0_% after 1 day and 7 days of nitrogen starvation. The five datasets were subjected to hierarchical clustering, resulting in seven distinct clusters representing similar phenotypic patterns. Thus, these seven clusters represent sets of gene deletions of genes encoding chromatin factors with similar quiescence phenotypes (Figure 4A,B and Figure S4). Investigation of the cluster profiles showed a weaker or miscellaneous phenotypic behavior in some of them (cluster 1, 6, and 7). Therefore, we focused the following analysis only on the gene clusters that were clearly required for maintenance of viability during quiescence (clusters 2 and 3) and for efficient quiescence entry into G_0_ (clusters 4 and 5). The details of all genes in the seven clusters are shown in Table S1.

#### 2.2.1. Chromatin Factors Required for Entry into G_0_

**Figure 5:**
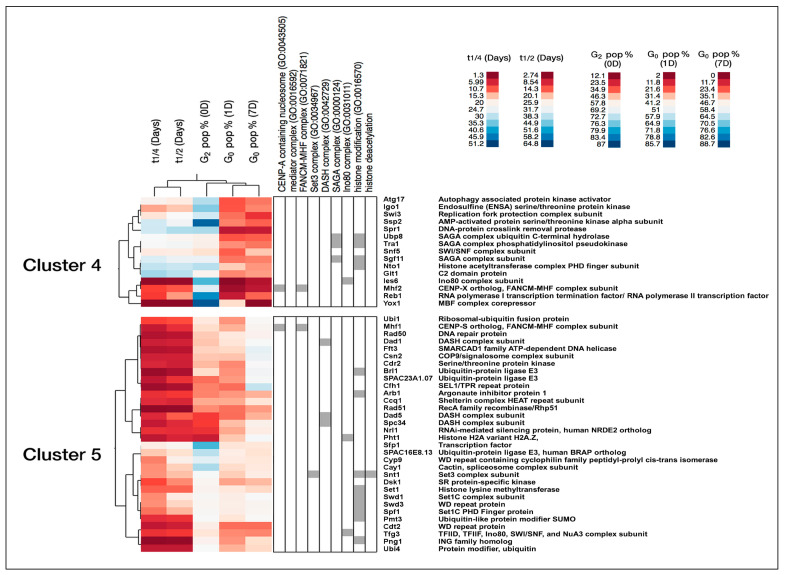
Characteristics of genes in clusters 4 and 5 being important for quiescence entry. Left: heat maps of the phenotypic clusters. The colors represent normalized data values (as indicated). The middle panel shows relevant Gene Ontology (GO) terms, and the annotated gene lists for each cluster are shown to the right.

Using the hierarchical clustering results, we first analyzed the step of entry into quiescence. It was clear that cluster 4, containing 15 genes, had a strong defect in the entry into G_0_ stage (Figure 4A,B). Interestingly, three genes, encoding Ubp8, Tra1, and Sgf11 subunits of the SAGA complex, were significantly enriched (15.2-fold enrichment, *p* = 6.4 × 10^−4^) in this cluster (Figure 5 and Table S2). This suggests an essential role for the SAGA complex in entry into quiescence (see Section 3).

Another phenotypic cluster showing milder defects in G_0_ entry step is cluster 5 with 32 genes. This gene cluster shows significant enrichment for the DASH protein complex (7.1-fold enrichment, *p* = 6.1 × 10^−3^) and the Set1C/COMPASS complex (4-fold enrichment, *p* = 1.2 × 10^−4^) that catalyzes the methylation at lysine 4 of histone H3 (Figure 5 and Table S2). This indicates that DASH and Set1C functions also are important at the G_0_ entry step. Another notable member of cluster 5, being important for G0 entry, is the gene encoding the nucleosome remodeling factor Fft3 (see Section 3).

#### 2.2.2. Chromatin Factors Required for Viability during Quiescence

Next, we used the clustering approach to investigate the chromatin regulatory gene functions needed for viability during G_0_. Clusters 2 and 3 showed a relatively high G_0_ percentage at day 1 and mild or strong mortality phenotypes (i.e., short T_1/2_ and T_1/4_ values) (Figure 4A,B and Figure S4). To examine the gene functions in these clusters in more detail, we used gene ontology analysis (Table S2). For cluster 2, we found six genes encoding proteins in the Clr6 histone deacetylase complex (the large Rpd3 complex of *S. pombe*) as being significantly enriched (1.8-fold enrichment, *p* = 4.6 × 10^−2^). Regarding cluster 3, we identified five genes encoding components of the Ino80 chromatin remodeling complex (3.2-fold enrichment, *p* = 1 × 10^−2^), four genes encoding subunits of the kinetochore associated DASH complex (2.8-fold enrichment, *p* = 3.2 × 10^−2^), and two genes encoding parts of the RNA-induced transcription silencing (RITS) complex (6.3-fold enrichment, *p* = 2.5 × 10^−2^) as being significantly enriched. Thus, these complexes are important for viability when cells are maintained in quiescence and not important for viability during the vegetative cell cycle or entry into quiescence. Gene deletion belonging to cluster 3 showed stronger mortality phenotypes compared to those in cluster 2 (Figure S4).

#### 2.2.3. A Unique Role for the Histone H3-Encoding Gene hht2

**Figure 6:**
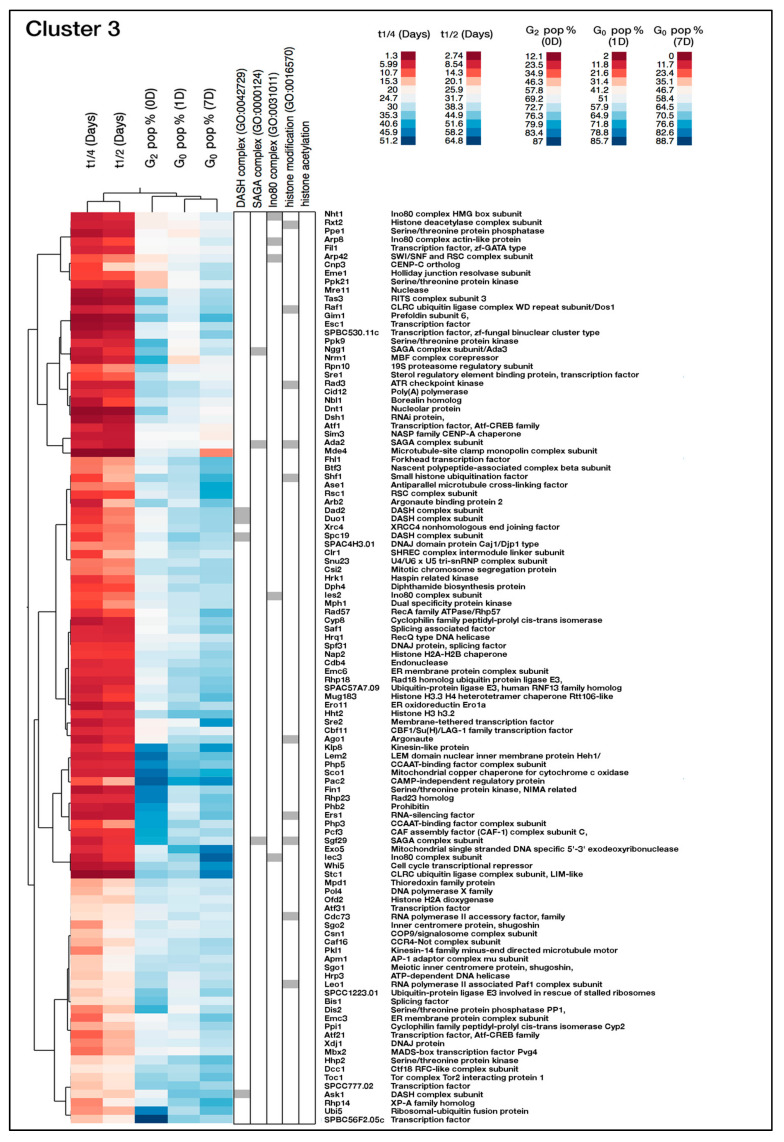
Characteristics of genes in cluster 3 being important for viability in G_0_. Left: heat maps of the cluster. The colors represent normalized data values (as indicated). The middle panel shows relevant Gene Ontology (GO) terms, and the annotated gene list is shown to the right.

Cluster 3 contains 108 genes with annotations listed in Figure 3. In addition to the complexes mentioned above, it is notable that only one of the histone H3-encoding genes, *hht2*, belongs to this cluster (Figure 6). Therefore, we examined all histone-encoding genes in our library and found that the gene deletion for *hht2* had a very strong mortality phenotype (T_1/2_ = 9.8 days), whereas the other gene deletions for histone H3 in *S. pombe*, *hht1* and *hht3,* showed only mild phenotypes and thus, *hht2* is the sole histone H3 gene essential for viability in G_0_ (Table S3).

### 2.3. Chromatin Regulatory Complexes Required for Viability during Quiescence

**Figure 7:**
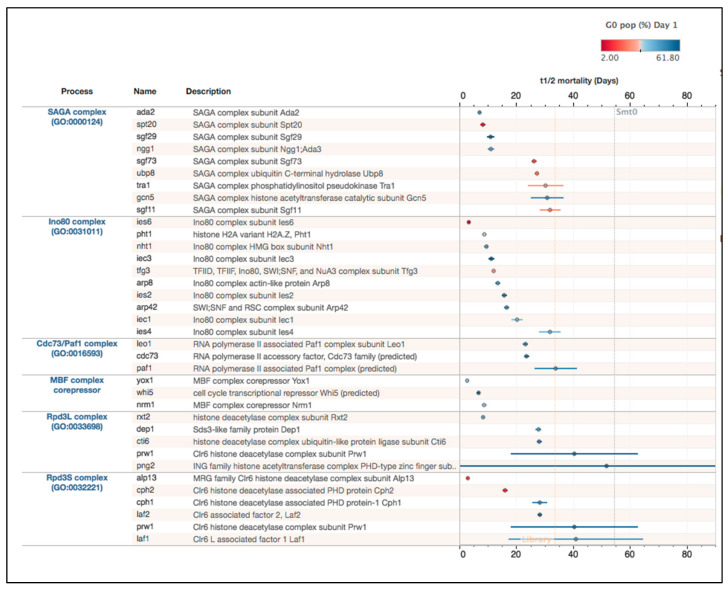
Comparison of G_0_ phenotypes for SAGA, INO80, Paf1, MBF corepressors, Rpd3L, and Rpd3S complexes. Left: Gene lists with annotations. Right: Half-time mortality T_1/2_ and dot color and circle size indicate the percentage of G_0_ cells. Horizontal lines indicate the 95% confidence interval. Smt0 = wild-type control.

To validate our dataset, we examined the measurements for gene deletions expected to be important for chromatin regulation in quiescence. As expected, gene deletions affecting Paf1 and Rpd3 complexes had a high G_0_ percentage and short T_1/2_ time (Figure 7). In the histone deacetylase (HDAC) complex annotated as Rpd3S (small) *alp13* and *cph2* genes are required for G_0_ entry, and mutants of the other subunits of both Rpd3S and Rpd3L (large) are to some extent required for survival in G_0_ (lower T_1/2_ time in comparison with Smt0 control in Figure 7).

We noticed that the gene deletion strains for Whi5 (a predicted Rpd3 corepressor) and its interacting proteins Yox1 and Nrm1, annotated as MBF corepressors, had very strong mortality phenotypes in early G_0_ (see Section 3).

Next, we examined the phenotypes of mutants affecting the SAGA and Ino80 complexes in more detail. Regarding the SAGA components, there was a dual phenotype with some genes being essential for efficient G_0_ entry (*spt20*, *sgf73*, *tra1*, *upb8*, *and sgf11*) and the other genes (*ada2, sgf29, ngg1*, and *gcn5*) being required for survival after the entry into G_0_. Regarding Ino80, although only 5 of 10 subunits were identified by the clustering approach described above (see cluster 3 in Figure S4), we found that all 10 subunits actually have quiescence-related phenotypes. Two Ino80 mutants, *ies6* and *tfg3,* are essential for G_0_ entry and mutants for the other eight Ino80 subunits had substantially shorter T_1/2_ times in comparison with the wild-type control (Figure 7). Thus, these observations suggest that both of these multisubunit complexes have distinct roles in quiescence entry and in later steps during quiescence.

#### 2.3.1. Validation of Mortality Phenotypes in G_0_ for Ino80 Mutants

Next, we performed two additional cell culture experiments to validate the mortality phenotypes for all 10 gene deletions affecting components of the Ino80 complex. We used cultures in biological duplicates and triplicates for each mutant, with *hht2* as positive control and the wild-type control (Smt0). The mortality was then measured by viability straining and flow cytometry at different time-points for up to two weeks of nitrogen starvation (Figure S5). Again, in both experiments, all 10 Ino80 mutants showed mortality phenotypes, with *ies6* and *pht1* showing the strongest and *ies4* the mildest phenotypes (see Figure S5 and Figure S7). Thus, it was clear that the Ino80 mortality phenotypes in G_0_ were highly reproducible with both experiments yielding very similar results as compared to the original high-throughput screen.

#### 2.3.2. Comparison of Swr1 and Ino80

We found a strong phenotype for the *pht1* gene encoding the histone variant H2A.Z in G_0_ (Figure 7). H2A.Z is deposited into chromatin by the Swr1 complex and removed by the Ino80 complex. To further understand the role of H2A.Z in quiescence, we next compared and contrasted the quiescence phenotypes for these two nucleosome remodeling complexes (Figure S6). As already demonstrated, it is clear that all Ino80 subunits have important functions in quiescence whereas a majority of Swr1 subunits are dispensable for survival (only *arp6* and *swc5* mutants have mortality phenotypes stronger than the library average). Thus, expression of H2A.Z from the *pht1* gene and deposition of H2A.Z by Ino80 seem to be more important than removal of H2A.Z by the Swr1 complex in quiescent cells (see Section 3).

### 2.4. DNA Repair Processes Required for Survival in Quiescence

**Figure 8:**
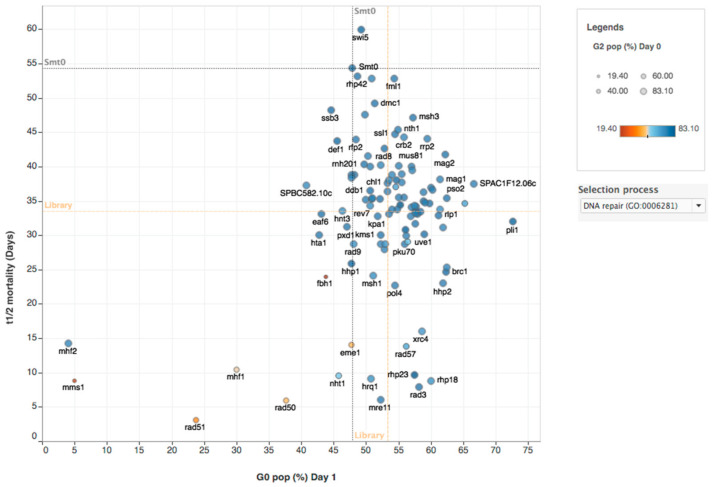
The phenotype of mutants affecting DNA repair during quiescence. Mutants with low survival (low T_1/2_ mortality rate) are located in the bottom side of graph (parameters: G0 entry and half-time mortality rate and G2% in T-0). Orange dashed lines indicate the respective median values of all mutants analyzed for each axis. Grey dashed lines indicate the value for *smt-0* wild-type strain for each axis.

Nondividing G_0_ cells are known to have different DNA damage patterns compared to proliferating cells, since G_0_ cells do not undergo DNA replication and other error-prone processes. Furthermore, in haploid G_0_ cells, some DNA repair processes requiring homology from a sister chromatid cannot take place. To get some insight into the repair processes, we analyzed the quiescence phenotypes of all genes in our dataset GO annotated as being DNA repair genes. We found that genes involved in several different DNA repair processes had strong mortality phenotypes in quiescence (Figure 8). Two genes (*mms1* and *rad51)* involved in homology-dependent repair (HR) and two genes (*mhf1* and *mhf2*) involved in double-strand break repair via synthesis-dependent strand annealing (SDSA) were found to be required for G_0_ entry. Seven genes encoding proteins in four distinct DNA repair pathways were clearly required for viability in G_0_: Rad50 and Mre11 are part of the MRN complex; Rad3 is a checkpoint protein, Xrc4 is involved in nonhomologous end-joining (NHEJ) and three proteins in the nucleotide excision repair (NER) pathway (Rhp23, Hrq1, and Rhp14) (See Section 3). This analysis also confirmed that Nht1, a DNA binding subunit of the Ino80 complex that is annotated as being involved in DNA repair, is important for viability in G_0_.

## 3. Discussion

### 3.1. Chromatin Regulation during Entry into Quiescence

When fission yeast cells are subjected to nitrogen starvation, they respond by exiting the cell cycle and activate the mating pathway to form zygotes that can enter into the meiotic program resulting in spore formation. However, when cells of the opposite mating type are absent, for example, in cultures of heterothallic (unswitchable) strains, the cells stop growing by elongation and instead rapidly divide twice to form small round cells that eventually become arrested in the quiescent G_0_ state with condensed nuclei and hyperclustered telomeres [3,14]. The G_0_ entry process has been shown to require several genes involved in different cellular functions, however, the regulation of chromatin during this process has hitherto been obscure.

Here we developed a high-throughput flow cytometry approach suitable for analysis of quiescent fission yeast cells. Although nuclear division in fission yeast occurs during mitosis, septum formation is delayed and occurs during S phase. Thus, cells in S phase sometimes have a DNA content higher than 2C, and in the G2 phase cells have a 2C DNA content [3]. Therefore, the lack of completed cytokinesis leads to the occurrence of single cell particles with two nuclei in G1 with total 2C DNA content (G1 is 1C DNA content). Hence, in vegetative fission yeast, both G1 (binuclear) and G2 (mononuclear) are detected in the 2C peak by conventional histogram analysis of flow cytometry, and cannot be distinguished from each other [3]. However, these two populations of cells can be distinguished by a specific gating strategy using DNA-A (total area of DNA signal) vs. DNA-W (DNA signal) [3]. On the other hand, during nitrogen starvation, a large number of cells die and can affect the accuracy of DNA content data, hence we used a dual staining strategy to discard dead cells before using DNA content analysis to study on G_0_ entry. In this case, mononuclear cells (G2) have a lower DNA signal (DNA-W) in comparison with binuclear cells (G1, M) in vegetative stage. Additionally, cells can be distinguished by the area of the DNA-A signals [15]. Therefore, in a DNA-A vs. DNA-W analysis, cells with total 1C DNA content (G0/arrested cells in G1) that are smaller and round shaped are found below the 2C DNA content cell population (G2 and G1) that contains bigger cells with rod shape. Thus, this strategy allowed us to simultaneously collect data for two parameters in quiescence, mortality and DNA content.

Our flow cytometry approach combined with hierarchical clustering identified two chromatin-modifying complexes as being essential for G_0_ entry in fission yeast (i.e., SAGA and Set1C). The SAGA complex is involved in histone acetylation and coactivation of transcription [15]. Set1C/COMPASS is required for methylation of histone H3 at lysine 4 (H3K4me), a histone mark generally involved in activation of transcription [16]. Thus, it is conceivable that the transcription of some genes needs to be activated by SAGA and Set1C to allow for G_0_ entry in fission yeast. Curiously, the SAGA complex was shown to be required for repression of genes involved in meiosis and sporulation in a heterothallic strain [17]. The mechanistic role of SAGA in gene repression is not clear but it is possible that this function also plays a role in G_0_ entry. One of the SAGA subunits needed for G0 entry is Tra1. This subunit has previously been attributed with a special function within SAGA having a specific in role affecting stress response genes and recruitment of SAGA to a subset of genes [18]. Hence, Tra1 could perform a similar function important in G_0_ entry at key genes.

Another protein complex that is required for efficient G_0_ entry is DASH. This complex plays a role at kinetochores in *S. pombe* [19]. Currently it is not clear how DASH contributes to the process of G_0_ entry although one possibility is that it is needed for chromosome segregation during the two rapid rounds of mitosis leading to G_0_ arrest.

It was recently suggested that clustering of telomeres is an important function in quiescent cells to keep the chromosome ends in a safe zone at the nuclear periphery [14]. We have earlier shown that subtelomeric chromatin boundary function and the association between telomeres and the nuclear envelope depend on Fft3 activity in vegetative cells [20]. Therefore, it is plausible that the quiescence defects we observed in *fft3* cells (i.e., inefficient G_0_ entry and high mortality) are related to a defect in telomere chromatin organization.

### 3.2. Chromatin Factors Essential for Viability during Quiescence

Fission yeast G_0_ cells can survive for several weeks in the absence of nitrogen and this requires autophagy and other metabolic adaptation processes [21,22]. The gene expression in G_0_ cells is reduced to about 20% compared to vegetative cells and ribosome biogenesis (including rRNA synthesis) is also largely shut down [5]. It is likely that this global shut down of transcription requires repression mechanisms acting on chromatin. Prior to this work, a role for the RNA interference pathway was shown to be essential for survival in G_0_ in fission yeast [7,8]. In this process, the Clr4 H3K9 lysine methyl-transferase is guided by small RNA, produced by the RNAi machinery, to silence the transcription of genes by formation of heterochromatin. Consistent with these observations, we identified components of the RITS complex as being essential for survival in G_0_. Our high-throughput approach also confirmed that genes encoding subunits of the Paf1 complex are required for viability during long-term quiescence [9].

In budding yeast cells, a global shutdown of gene expression occurs in quiescent cells by histone deacetylation carried out by the Rpd3 complex at gene promoters [12]. Our results pointed out the fission yeast Clr6 complex having a vital role in quiescence. Thus, histone deacetylation is a conserved function in quiescence now evident in two different eukaryotic model organisms. Both in budding yeast and metazoans, the expression of genes involved in the G1/S transition is activated by transcription factors, which need to be counteracted by corepressors and Rpd3-like histone deacetylase complexes for entry into G_0_ [23]. In *S. pombe*, the G1/S transition is regulated by MBF (MluI cell cycle box binding factor) and is counteracted by the Yox1 repressor protein that inhibits the transcription of MBF target genes [24]. Interestingly, our data establish a role for Yox1 in G_0_ along with Whi5 and Nrm1 also being annotated as MBF-corepressor proteins. The phenotype of the MBF corepressor mutants is a very short T_1/2_ survival time in G_0_ (<10 days). Therefore, we hypothesize that MBF target genes need to be continuously repressed by MBF corepressors in G_0_ to maintain viability of the cells. We also propose that Rpd3 complexes containing the HDAC Clr6 could be involved in this gene repression function.

Perhaps the most striking observation of this study was that all 10 tested gene deletions affecting subunits of the Ino80 complex have strong quiescence-related phenotypes. Ino80 is a conserved nucleosome remodeling complex with an ATP-driven motor activity that enables histone exchange from H2A.Z to H2A [25,26]. The fission yeast Ino80 complex has been shown to evict nucleosomes in vitro and control gene expression by nucleosome eviction in vivo [27]. Interestingly, Ino80 interacts with MBF in fission yeast and plays a role in activation of genes driving G1/S transition of the cell cycle [28]. Our findings shed light on a novel function of Ino80 during cellular quiescence. We suggest that Ino80 is required for proper gene regulation in cells during G_0_ entry and at later time points in G_0_. Ino80 could also be implicated in some aspect of DNA damage repair in G_0_ [29]. Another important function of Ino80 that was recently discovered in fission yeast is transcription-coupled histone H3 turnover [30]. In this context, we observe that one gene encoding histone H3, *hht2+*, is essential in G_0_. Interestingly, this gene has previously been shown to be the major contributor to histone H3 expression compared to the two other histone H3-encoding genes in *S. pombe* [31]. Thus, it is conceivable that histone H3 turnover driven by Ino80 and *hht2+* is essential for viability of G_0_ cells.

To further understand the role of H2A.Z, we compared phenotypes of gene deletions affecting the Swr1 complex with those affecting Ino80. Swr1 is a conserved nucleosome remodeling complex required for the deposition of H2A.Z into chromatin [32]. Swr1 is required for H2A.Z deposition also in fission yeast [33]. It was clear that, in sharp contrast to Ino80 mutants, most Swr1 mutants do not show any phenotype in G_0_. Therefore, we conclude that the removal of H2A.Z, possibly linked to gene activation or DNA repair, is more important than the process of H2A.Z deposition in G_0_ cells.

### 3.3. DNA Repair in G0

It has been reported that fission yeast G_0_ cells have different DNA damage sensitivities and responses compared to proliferating cells [34]. For example, G_0_ cells are hypersensitive to UV radiation and gamma rays compared to vegetative cells. It was shown that the ATR-like checkpoint-kinase Rad3 and the Ku80 protein involved in the NHEJ pathway are essential in G_0_ after irradiation by UV [34]. The same study revealed that repair of double-strand breaks after UV or gamma irradiation in G_0_ cells requires Ku80. A recent study demonstrated that fission yeast G_0_ cells accumulate mutations spontaneously as a linear function of time. Both small deletions and single nucleotide mutations are increased over time [35]. It is possible that these damages are increased due to the inability of G_0_ cells to undergo DNA repair by homologous recombination (HR) using a sister chromatid. Prior to this work it was shown that the Tdp1 protein, involved in single-strand break DNA repair together with topoisomerase I, is essential in G_0_ cells [36]. Here we show a requirement for Rad50 and Mre11 in G_0_ cells. Both of these proteins are part of the MRN complex (Mre11, Nbs1, and Rad50). The role of MRN is to process double-strand breaks and to activate the ATM kinase before repair by HR or NHEJ [37]. Our results show that the ATM-like Rad3 checkpoint protein is essential for survival in G_0_, even in the absence of irradiation. This is consistent with the finding that G_0_ cells accumulate DNA damage over time [35]. We also demonstrate that Xrc4, a member of the NHEJ pathway, and three proteins in the nucleotide excision repair (NER) pathway (Rhp23, Hrq1, and Rhp14) are essential in G_0_. NER was demonstrated to occur in fission yeast G_0_ cells using antibodies that detect thymidine dimers that were readily removed after UV irradiation [34]. Our results suggest that NER is an important DNA damage repair mechanism for spontaneous damage in quiescent cells. To summarize, we find that several DNA repair pathways are crucial to handle the spontaneous damage that occurs in G_0_ cells: the MRN complex, Rad3, nonhomologous end-joining, and nucleotide excision DNA repair.

## 4. Materials and Methods

### 4.1. Preparation of Gene Deletion Library in Prototrophic Strains

The fission yeast haploid gene deletion library version 5.0 was obtained from the Bioneer coorporation, Daejeon, Republic of Korea [13]. This library contains a collection of 3420 haploid gene deletion mutants (covering about 92% of all nonessential genes) contained in 36 plates in 96-format arrays. The auxotrophic l*eu1*-32 *ade6*-M216/M210 *ura4*-D18 markers of the original deletion library is unsuitable to analyze fission yeast in quiescence under nitrogen-depleted conditions. To remove all auxotrophic markers, we crossed out all the strains in the Bioneer v. 5.0 library to obtained a prototrophic gene deletion library following the procedure described by [6]. Briefly, using the RoToR robot (Singer Instruments, Somerset, UK), mutants from the haploid Bioneer v. 5.0 library were crossed with the *mat1-M smt-0* wild-type strain using SPA media plates [38], left to sporulate at 25 °C for 48 h. The plates were then incubated at 42 °C during 72 h to eliminate vegetative cells. Next, spores were transferred to yeast extract with supplements (YES) semi-solid media to germinate for 48 h. Plates with mutants were successively spotted three times on semi-solid Edinburgh Minimal Medium (EMM) without leucine, adenine, and uracil sources to select prototrophic mutants and then on YES medium containing G418 (150 ug/mL) to select for the kanMX4 cassette used for gene deletions. Altogether, three rounds of EMM2 without leucine, adenine, and uracil and YES + G418 selection were performed. The generated prototrophic gene deletion library of 3364 mutants was then stored at –80 °C in YES media containing 40% glycerol. For the present study, 740 mutant strains harboring deletions of genes involved in chromatin or transcription regulation processes were selected from the generated prototroph library using *S. pombe* Gene Ontology terms such as “chromatin binding”, “DNA binding”, “chromosome binding”, “chromosome”, and “transcription”. Mutants were selected manually using Gene Ontology and displayed in eight plates of 96-well format. Prototroph mutants were checked using DNA content using flow cytometry of proliferating mutant cells resulting in the exclusion of 55 diploid mutants.

### 4.2. Preparation of Vegetative and Quiescence Cells Samples

Most of the library manipulations were performed with the RoToR robot (Singer) except for liquid handling that used automatic multipipettes channels. The frozen library was stored in 96-well plates, thawed, and inoculated in 96-array format on semi-solid YES media. Plates were incubated for 2–3 days at 30 °C.

Using RoToR, small amounts of cell culture were next transferred using long pin pad (Singer) to 96-well plates containing 200 μL of liquid YES media per well. Plates were next sealed with a hydrophobic porous sealing film and incubated in a humid chamber inside a shaking incubator at 30 °C, at 200 rpm for about 9–10 h. Cultures from the largest colonies previously grown on semi-solid YES media plates were verified using a hemocytometer to have a cell concentration between 10^+6^ and 10^+7^ cells/mL. Cells in 96-well plates were then pelleted by centrifugation at 400× *g* for 5 min, washed twice, and resuspended with 200 μL of prewarmed EMM media at 30 °C. Next, 3 μL cell culture was transferred into 96-well deep well plates of 2 mL and supplemented with 1.5 mL of fresh prewarmed EMM media at 30 °C. Plates were then sealed with hydrophobic porous sealing film and put in a humid chamber and incubated in a shaking incubator at 200 rpm, 30 °C for 12–16 h. Cell cultures in plates were then verified again to reach a concentration of 10^+6^ to 10^+7^ cells/mL. For cell samples with nitrogen source (Day 0), a volume of 30 μL of cell culture from each well was taken from 96-well plates. For cell samples cultivated in nitrogen-depleted media, cells in 96-well deep well plates were centrifuged at 400× *g*, 5 min and washed twice with 200 μL of prewarmed EMM media without nitrogen (EMM-N_2_). Cell pellets were resuspended in 1.5 mL of fresh prewarmed EMM-N2 media and 96-well deep well plates were sealed with hydrophobic porous sealing film and placed in a humid chamber, incubated at 30 °C, 200 rpm. Aliquots of 30 μL were taken for day 1, 7 and 50 μL for day 14, 21, 28 were taken from cell cultures. Each sample collected using 96 well-plates were kept on ice for cytometry cell preparation. Two independent biological replicates of the library were measured.

### 4.3. Preparation of Cells for High-Throughput Flow Cytometry

Cells from 96-well plates were pelleted for 5 min at 400 g, 4 °C and supernatants were discarded. Each well was supplied with 150 μL of Live-or-Dye™ Fixable Viability Staining λ_Ex_/λ_Em_ 642/662 nm (Biotium, Fremont, CA, USA) diluted at 1/1000^e^ in phosphate-buffered saline (PBS) using a multichannel pipette and incubated 30 min in the dark on ice. Next, 200 μL of PBS was added in each well. Plates were next centrifuged at 4500× *g* for 5 min and supernatants were discarded. Cells were resuspended in a volume of 100 μL of 70% ethanol on ice to permeabilize cell wall for DNA staining. Cells were incubated for 30 min at 4 °C. Cells in 96-well plates were twice incubated with 200 μL of 50 mM sodium citrate, pH 7 for 10 min and washed at 4500× *g* for 10 min. Cells were then resuspended with 200 μL sodium citrate (50 mM sodium citrate, pH 7.0) containing 0.2 mg/mL RNase A DNase-inactivated (Roche diagnostics Scandinavia, Solna, Sweden, 10109169001) and incubated for 3 h at 37 °C. A volume of 200 μL PBS was added per well, cells were resuspended using multichannel pipette to remove cell aggregates, and plates were centrifuged at 4500× *g* for 10 min. Each volume of 100 μL of PBS with 5 μg/mL propidium iodide (PI) (Invitrogen AB, Stockholm, Sweden, P4864) was added per well. PI stains DNA and RNA and is not a membrane-permeable dye. We used it for investigation of both mortality, since it stains dead cells without fixation, and DNA content [15]. Plates were incubated in the dark at room temperature for 30 min. Samples were stored on ice and protected from light before analysis.

### 4.4. High-Throughput Flow Cytometry Measurement

Before analysis, 100 μL of PBS was added per well and 96-well plates were immediately analyzed using the multiplex flow-cytometer CytoflexS (Beckman Coulter, Bromma, Sweden) with CytExpert software (www.mybeckman.se/flow-cytometry/instruments/cytoflex/software). Briefly, samples were analyzed using slow running mode with a maximum of 300 cell count per second and about 20,000 events where counted in the living cell population. Cells were sorted by forward (FSC) and side (SSC) light scattering to identify single cells. Dead cells stained for free intracellular amines of the dead cells with Live-or-Dye™ Fixable Viability Staining λEx/λEm 642/662 nm (Biotium) were counted through the FSC-A vs FL3A (R660) channels (FL3A::660A). A660 signals + and – demonstrate dead cells and live cells, respectively. DNA content analysis was performed on the living cell population with propidium iodide, mononuclear G_2_ (2C1N), and mononuclear G_0_ (1C1N) cell populations were detected through the total area of DNA signal (DNA-A) vs. DNA signal (DNA-W) [11] and compared to the total amount of living cell population. A minimal cut-off of 1000 single cells was considered for each sample measurement.

### 4.5. Data Analysis Issued from High-Throughput Flow Cytometry Measurement

FlowJo software (https://www.flowjo.com/solutions/flowjo/downloads) was used to extract flow cytometry data measurement. Raw data were then compiled and analyzed using Excel (Microsoft), Tableau (Tableau; www.tableau.com), JMP (SAS) software (www.jmp.com) was used for data visualization and statistical analysis. To determined half-time mortality, nonlinear regression models were performed using JMP (SAS). Seven nonlinear models have been applied (Logistic 3P, Mechanistic Growth, Gompertz 3P, Logistic 5P, Exponential 3P, Exponential 2P, Probit 4P). Summary of the different tested models are presented in Figure S3. The optimal nonlinear model has been determined among the seven nonlinear regression models based on two conditional criteria: (1) overall highest Z score fitting compared to the other models, (2) the optimal nonlinear model was able to predict by nonlinear regression the given day for 99% mortality. When applicable, independent samples *t*-tests were applied to compare the *smt-0* control strain and mutants. Hierarchical clustering of mutant fission yeast strain phenotypes was performed with Ward’s method using the JMP software v.13.2.0 (SAS). The Cubic clustering criterion was used to estimate the optimal number of clusters (i.e., n = 7 in this study).

### 4.6. Gene Onthology (GO) Analysis

The obtained data was analyzed using GO slim categories from *S. pombe* recourse website (https://www.pombase.org). The overall analysis of our collection is shown in the Table S1.

## Supplementary Materials

Supplementary materials can be found at https://www.mdpi.com/1422-0067/21/23/9022/s1. Figure S1: Description of cell culture condition for high-throughput cytometry analysis, Figure S2: Detailed workflow of cell staining for flow cytometry analysis, Figure S3: The of optimal non-linear models that best fit the G0 mortality phenotypes in the library of gene deletions, Figure S4: Box plots representations of phenotype values for each gene cluster, Figure S5: Validation of Ino80 mortality phenotypes in G0, Figure S6: A comparison of mutant phenotypes in G0 for components of the Ino80 and Swr1 complexes, Table S1: MDYZ library Data, Table S2: Enrichment for GO slim ontology, complexes and type of histone modification per cluster, Table S3: A comparison of quiescence phenotypes for deletions of genes encoding histone H3.
