# Supplementary material for: High-Throughput Flow Cytometry Combined with Genetic Analysis Brings New Insights into the Understanding of Chromatin Regulation of Cellular Quiescence
Source: Int J Mol Sci. 2020 Nov 27;21(23):9022. doi: 10.3390/ijms21239022 (PMC7729564; doi:10.3390/ijms21239022)
Supplement: Supplementary file 1 [file ijms-21-09022-s001.zip › IJMS_995869_revised_Suppl_data_proof/IJMS_995869_revised_ Suppl data file.pptx]

## Slide 1
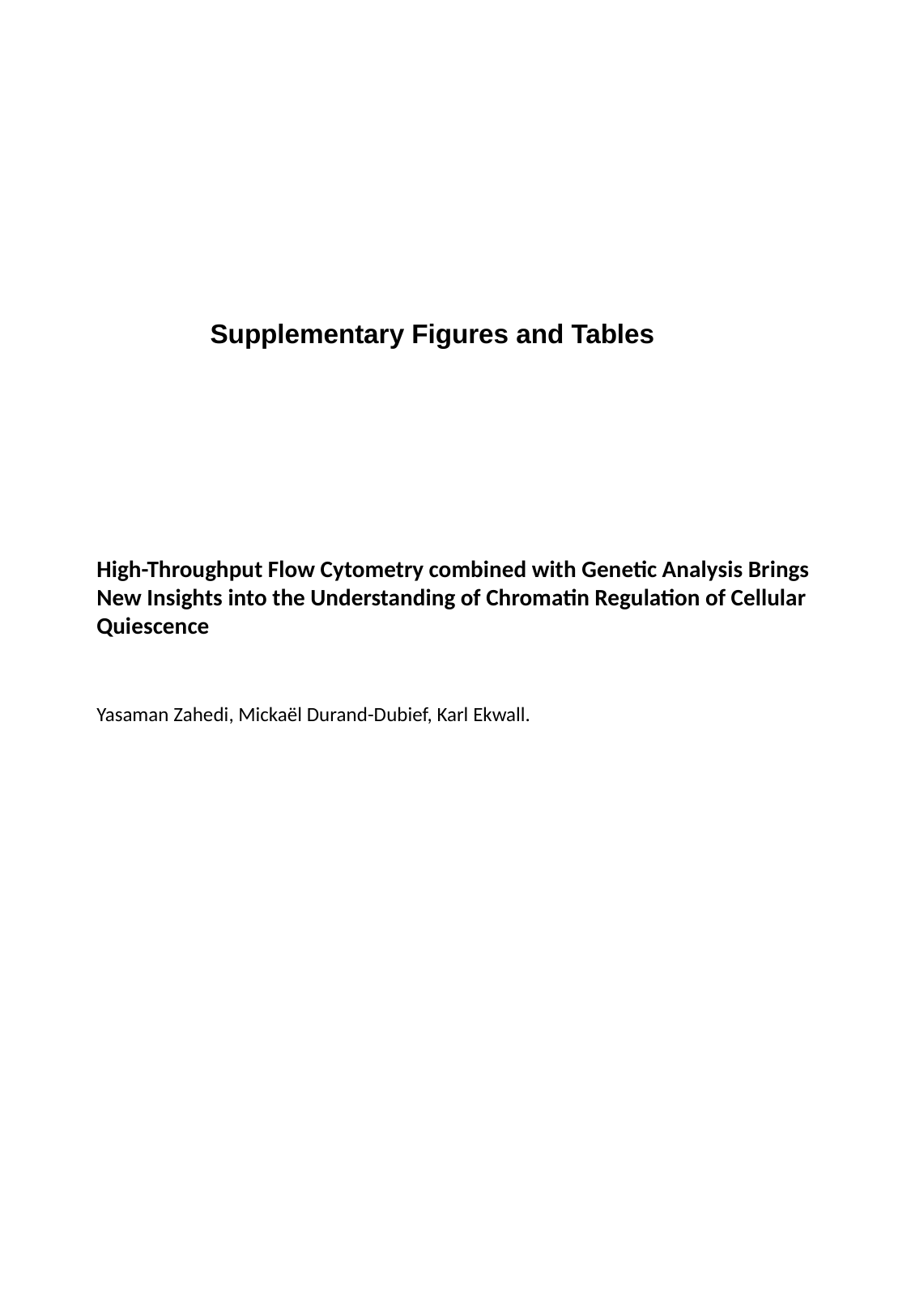

Supplementary Figures and Tables
High-Throughput Flow Cytometry combined with Genetic Analysis Brings New Insights into the Understanding of Chromatin Regulation of Cellular Quiescence
Yasaman Zahedi, Mickaël Durand-Dubief, Karl Ekwall.

## Slide 2
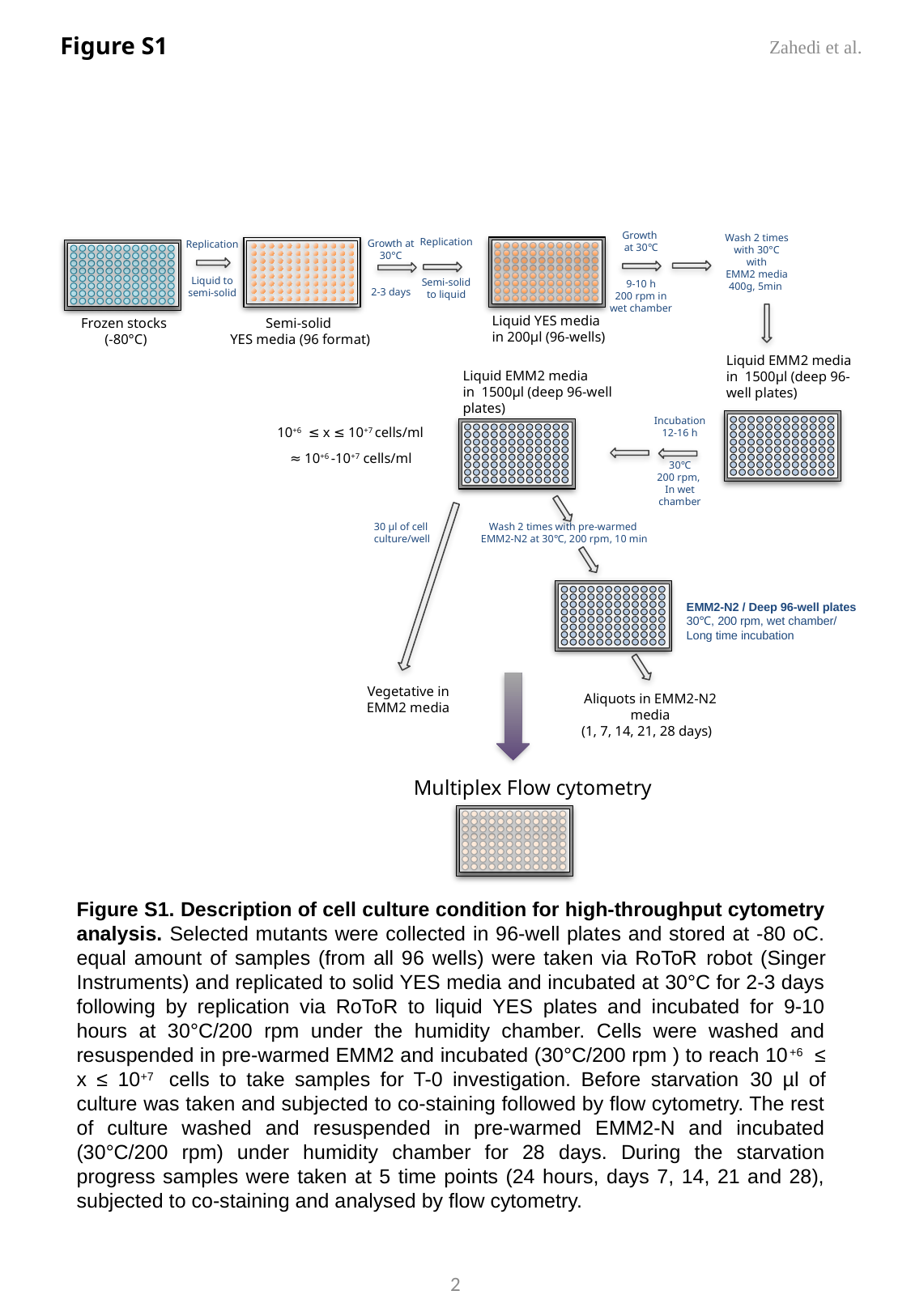

Zahedi et al.
Figure S1
Growth
at 30℃
9-10 h
200 rpm in wet chamber
Wash 2 times
 with 30°C
with
EMM2 media
400g, 5min
Replication
Semi-solid
to liquid
Growth at 30°C
2-3 days
Replication
Liquid to semi-solid
Liquid YES media
in 200µl (96-wells)
Frozen stocks
 (-80°C)
Semi-solid
YES media (96 format)
Liquid EMM2 media
in 1500µl (deep 96-well plates)
Liquid EMM2 media
in 1500µl (deep 96-well plates)
Incubation 12-16 h
30℃
200 rpm,
In wet
chamber
10+6 ≤ x ≤ 10+7 cells/ml
≈ 10+6 -10+7 cells/ml
30 µl of cell culture/well
Wash 2 times with pre-warmed
 EMM2-N2 at 30℃, 200 rpm, 10 min
EMM2-N2 / Deep 96-well plates
30℃, 200 rpm, wet chamber/ Long time incubation
Vegetative in EMM2 media
Aliquots in EMM2-N2 media
(1, 7, 14, 21, 28 days)
Multiplex Flow cytometry
Figure S1. Description of cell culture condition for high-throughput cytometry analysis. Selected mutants were collected in 96-well plates and stored at -80 oC. equal amount of samples (from all 96 wells) were taken via RoToR robot (Singer Instruments) and replicated to solid YES media and incubated at 30°C for 2-3 days following by replication via RoToR to liquid YES plates and incubated for 9-10 hours at 30°C/200 rpm under the humidity chamber. Cells were washed and resuspended in pre-warmed EMM2 and incubated (30°C/200 rpm ) to reach 10+6 ≤ x ≤ 10+7 cells to take samples for T-0 investigation. Before starvation 30 µl of culture was taken and subjected to co-staining followed by flow cytometry. The rest of culture washed and resuspended in pre-warmed EMM2-N and incubated (30°C/200 rpm) under humidity chamber for 28 days. During the starvation progress samples were taken at 5 time points (24 hours, days 7, 14, 21 and 28), subjected to co-staining and analysed by flow cytometry.
2

## Slide 3
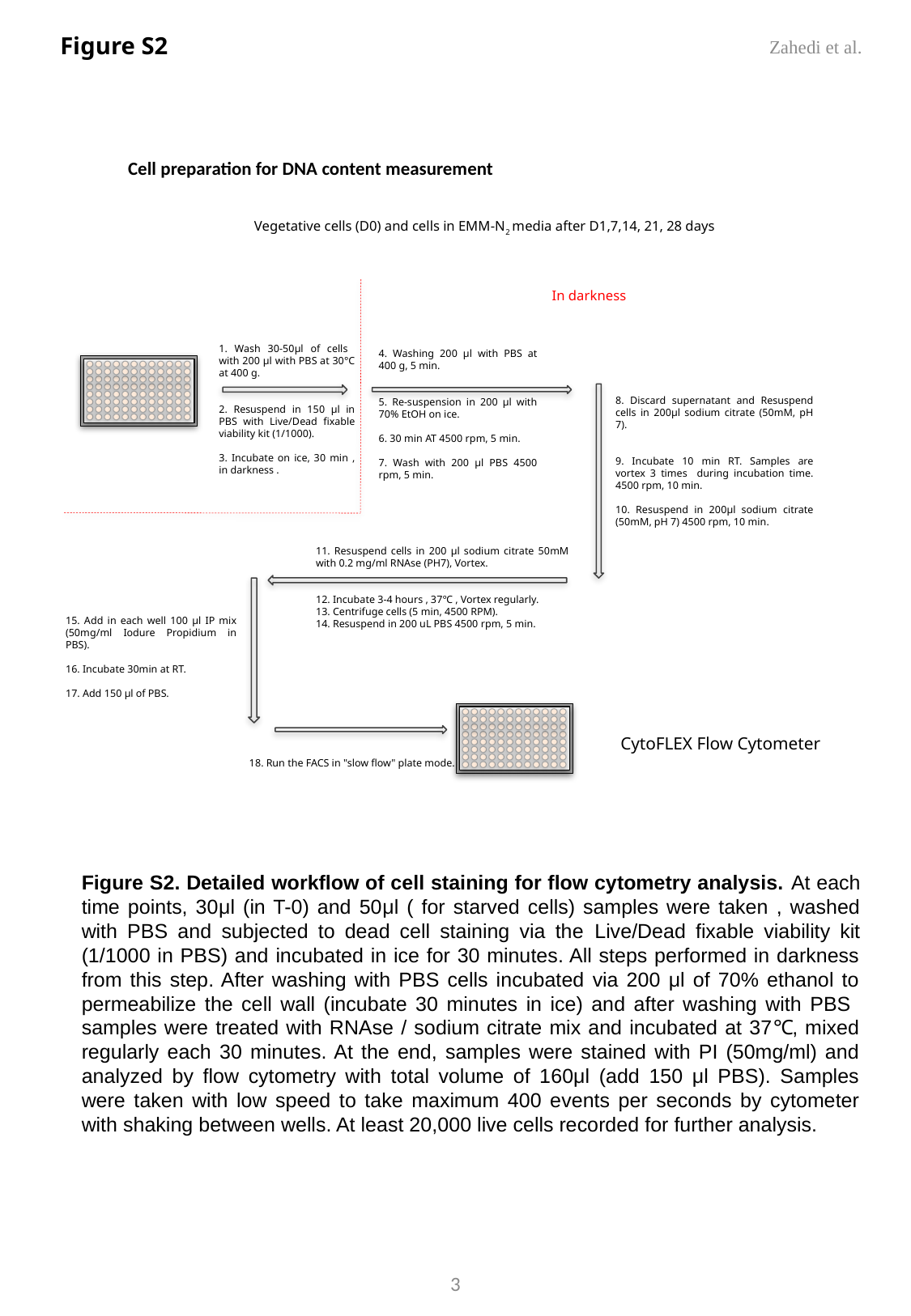

Zahedi et al.
Figure S2
# Cell preparation for DNA content measurement
Vegetative cells (D0) and cells in EMM-N2 media after D1,7,14, 21, 28 days
In darkness
1. Wash 30-50μl of cells with 200 μl with PBS at 30°C at 400 g.
2. Resuspend in 150 μl in PBS with Live/Dead fixable viability kit (1/1000).
3. Incubate on ice, 30 min , in darkness .
4. Washing 200 μl with PBS at 400 g, 5 min.
5. Re-suspension in 200 μl with 70% EtOH on ice.
6. 30 min AT 4500 rpm, 5 min.
7. Wash with 200 μl PBS 4500 rpm, 5 min.
8. Discard supernatant and Resuspend cells in 200μl sodium citrate (50mM, pH 7).
9. Incubate 10 min RT. Samples are vortex 3 times during incubation time. 4500 rpm, 10 min.
10. Resuspend in 200μl sodium citrate (50mM, pH 7) 4500 rpm, 10 min.
11. Resuspend cells in 200 μl sodium citrate 50mM with 0.2 mg/ml RNAse (PH7), Vortex.
12. Incubate 3-4 hours , 37℃ , Vortex regularly.
13. Centrifuge cells (5 min, 4500 RPM).
14. Resuspend in 200 uL PBS 4500 rpm, 5 min.
15. Add in each well 100 μl IP mix (50mg/ml Iodure Propidium in PBS).
16. Incubate 30min at RT.
17. Add 150 μl of PBS.
CytoFLEX Flow Cytometer
18. Run the FACS in "slow flow" plate mode.
Figure S2. Detailed workflow of cell staining for flow cytometry analysis. At each time points, 30μl (in T-0) and 50μl ( for starved cells) samples were taken , washed with PBS and subjected to dead cell staining via the Live/Dead fixable viability kit (1/1000 in PBS) and incubated in ice for 30 minutes. All steps performed in darkness from this step. After washing with PBS cells incubated via 200 μl of 70% ethanol to permeabilize the cell wall (incubate 30 minutes in ice) and after washing with PBS samples were treated with RNAse / sodium citrate mix and incubated at 37℃, mixed regularly each 30 minutes. At the end, samples were stained with PI (50mg/ml) and analyzed by flow cytometry with total volume of 160μl (add 150 μl PBS). Samples were taken with low speed to take maximum 400 events per seconds by cytometer with shaking between wells. At least 20,000 live cells recorded for further analysis.
3

## Slide 4
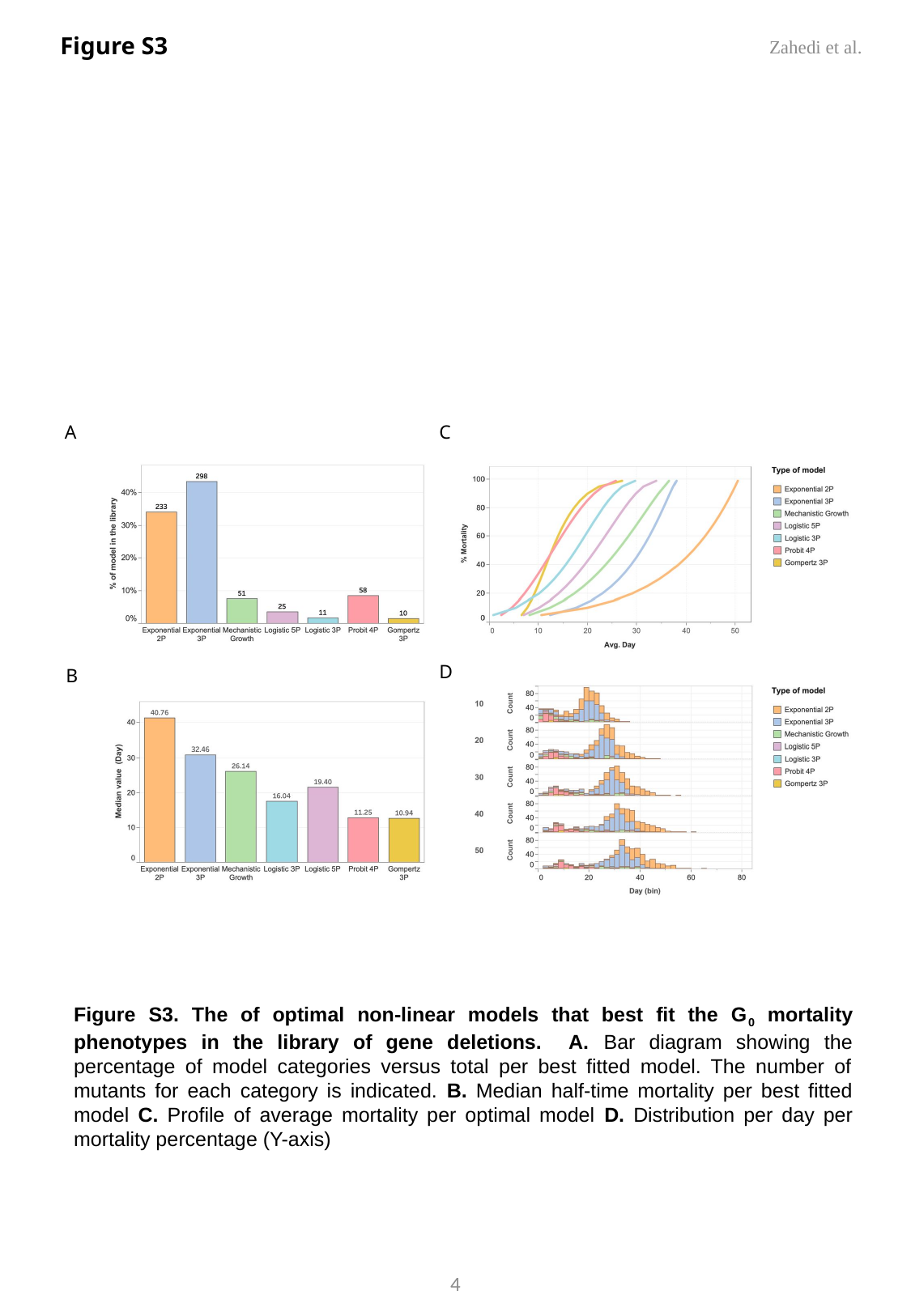

Zahedi et al.
Figure S3
A
C
D
B
Figure S3. The of optimal non-linear models that best fit the G0 mortality phenotypes in the library of gene deletions. A. Bar diagram showing the percentage of model categories versus total per best fitted model. The number of mutants for each category is indicated. B. Median half-time mortality per best fitted model C. Profile of average mortality per optimal model D. Distribution per day per mortality percentage (Y-axis)
4

## Slide 5
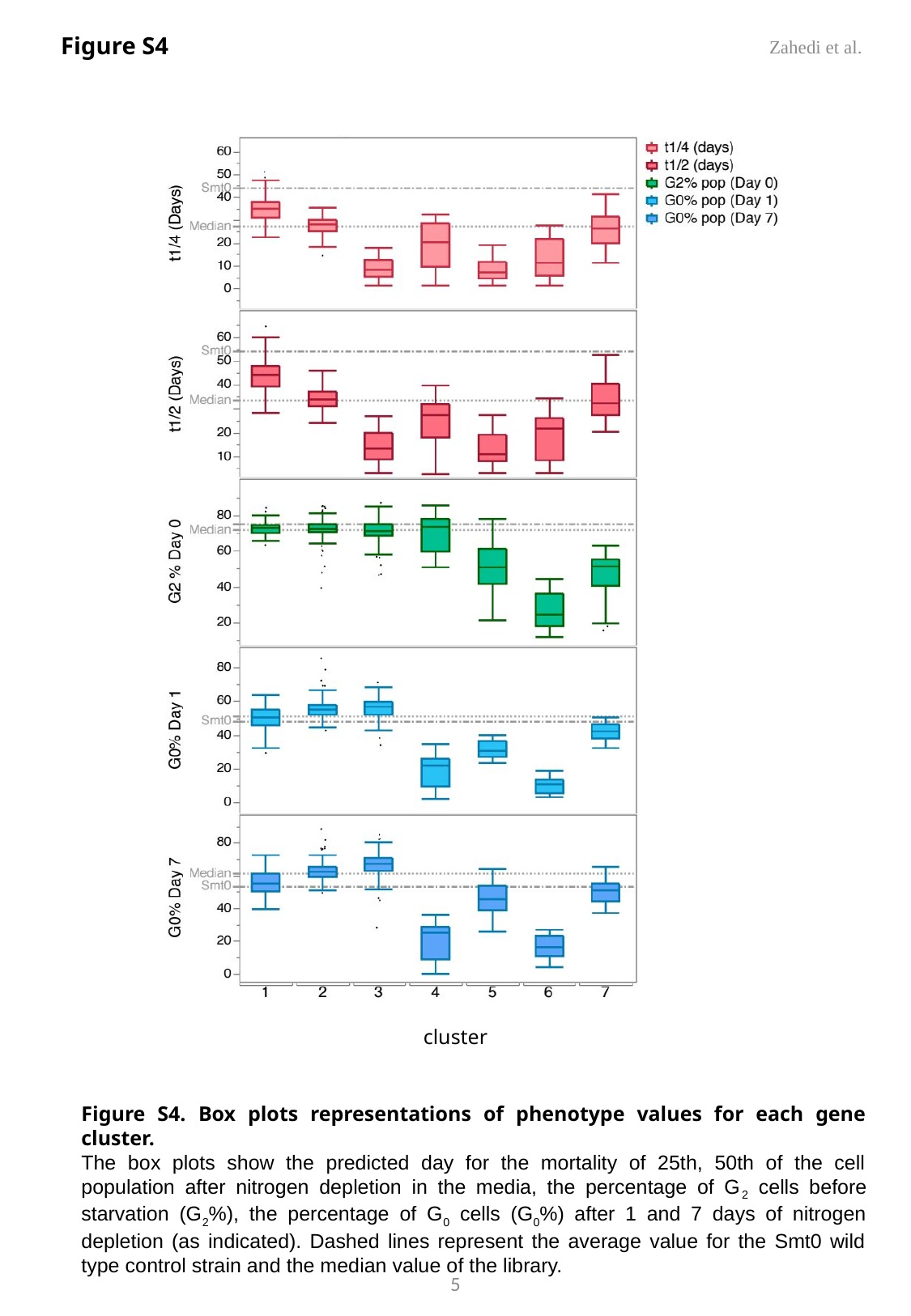

Zahedi et al.
Figure S4
cluster
Figure S4. Box plots representations of phenotype values for each gene cluster.
The box plots show the predicted day for the mortality of 25th, 50th of the cell population after nitrogen depletion in the media, the percentage of G2 cells before starvation (G2%), the percentage of G0 cells (G0%) after 1 and 7 days of nitrogen depletion (as indicated). Dashed lines represent the average value for the Smt0 wild type control strain and the median value of the library.
5

## Slide 6
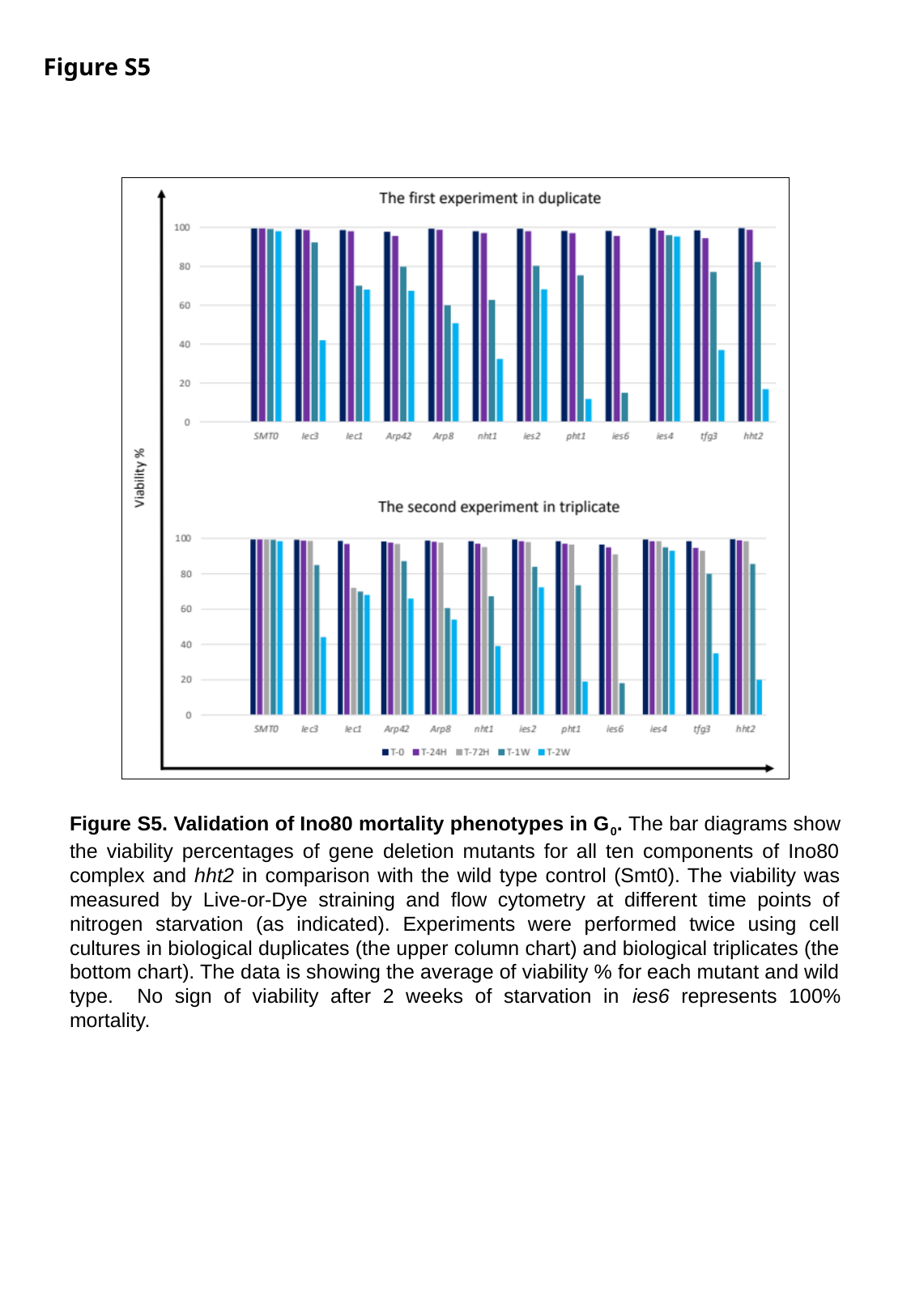

Figure S5
Figure S5. Validation of Ino80 mortality phenotypes in G0. The bar diagrams show the viability percentages of gene deletion mutants for all ten components of Ino80 complex and hht2 in comparison with the wild type control (Smt0). The viability was measured by Live-or-Dye straining and flow cytometry at different time points of nitrogen starvation (as indicated). Experiments were performed twice using cell cultures in biological duplicates (the upper column chart) and biological triplicates (the bottom chart). The data is showing the average of viability % for each mutant and wild type. No sign of viability after 2 weeks of starvation in ies6 represents 100% mortality.

## Slide 7
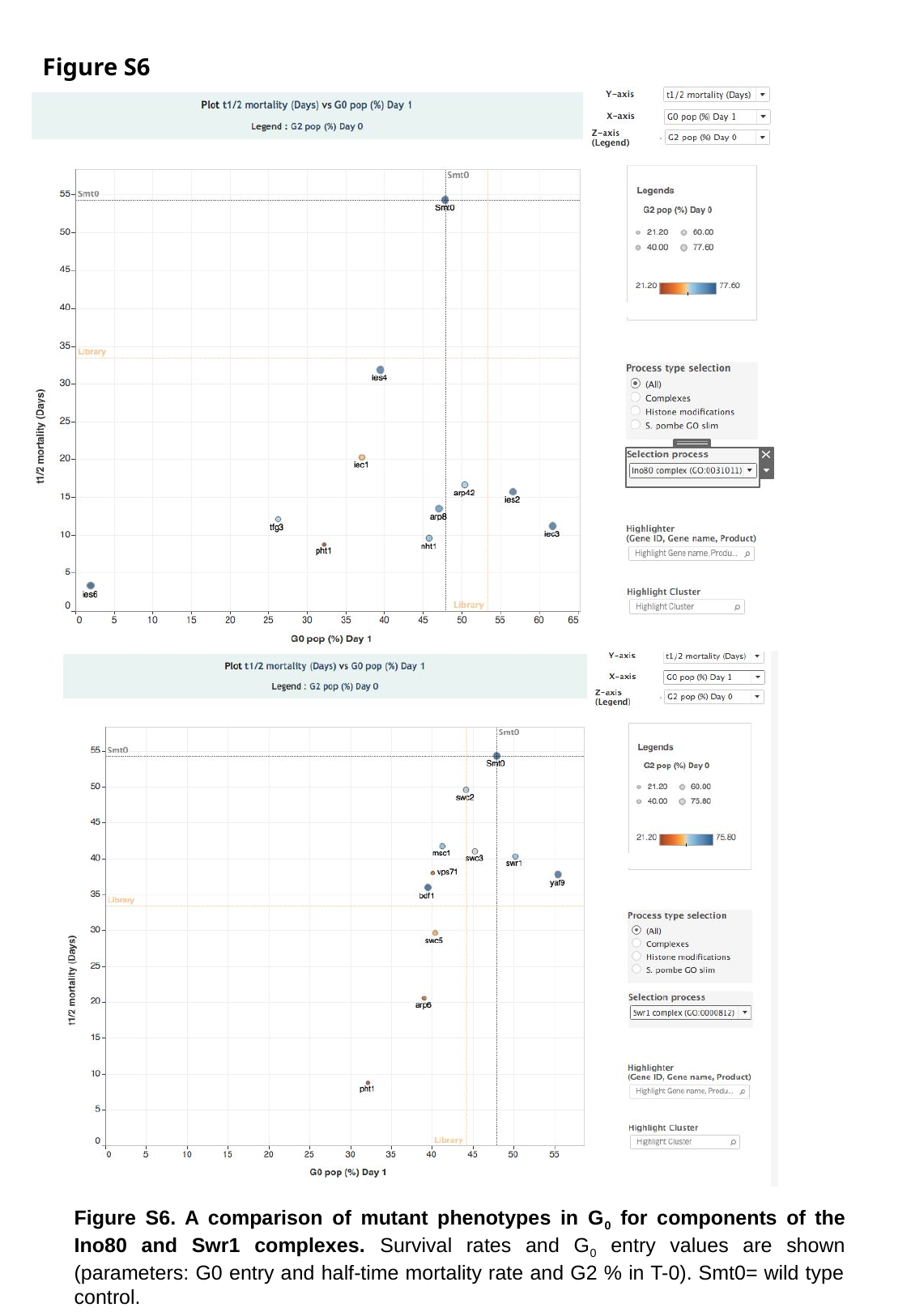

Figure S6
Figure S6. A comparison of mutant phenotypes in G0 for components of the Ino80 and Swr1 complexes. Survival rates and G0 entry values are shown (parameters: G0 entry and half-time mortality rate and G2 % in T-0). Smt0= wild type control.

## Slide 8
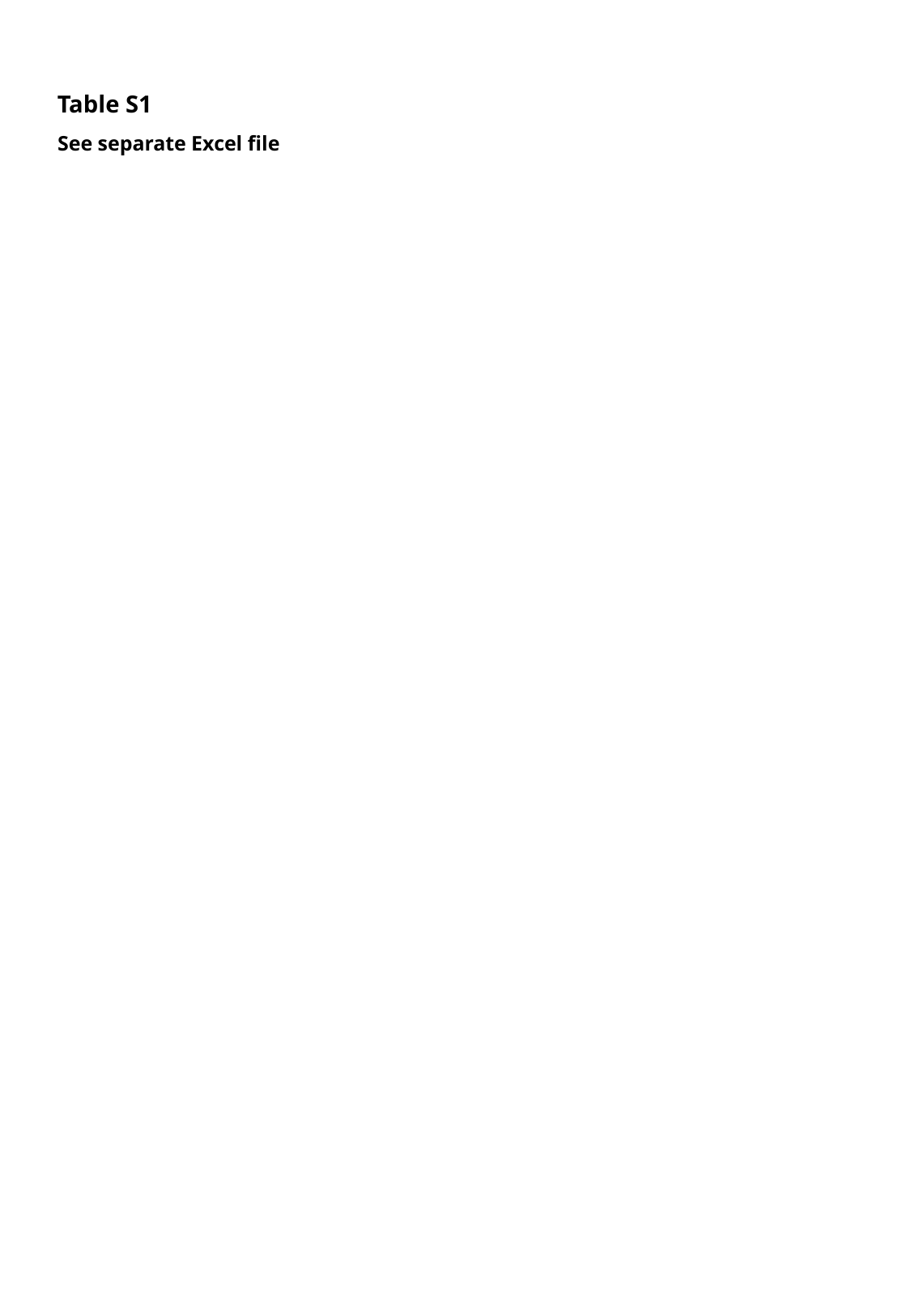

Table S1
See separate Excel file

## Slide 9
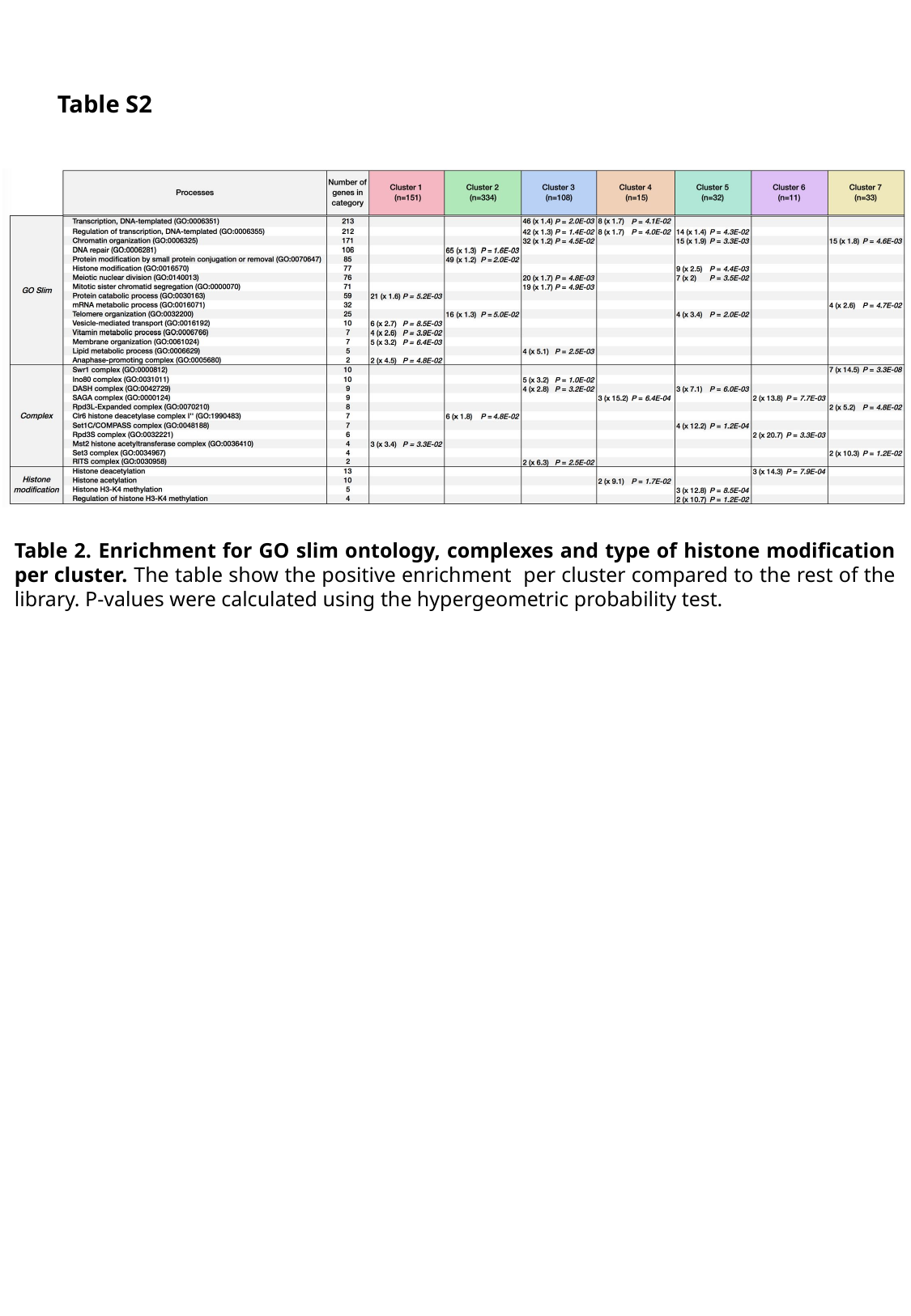

Table S2
Table 2. Enrichment for GO slim ontology, complexes and type of histone modification per cluster. The table show the positive enrichment per cluster compared to the rest of the library. P-values were calculated using the hypergeometric probability test.

## Slide 10
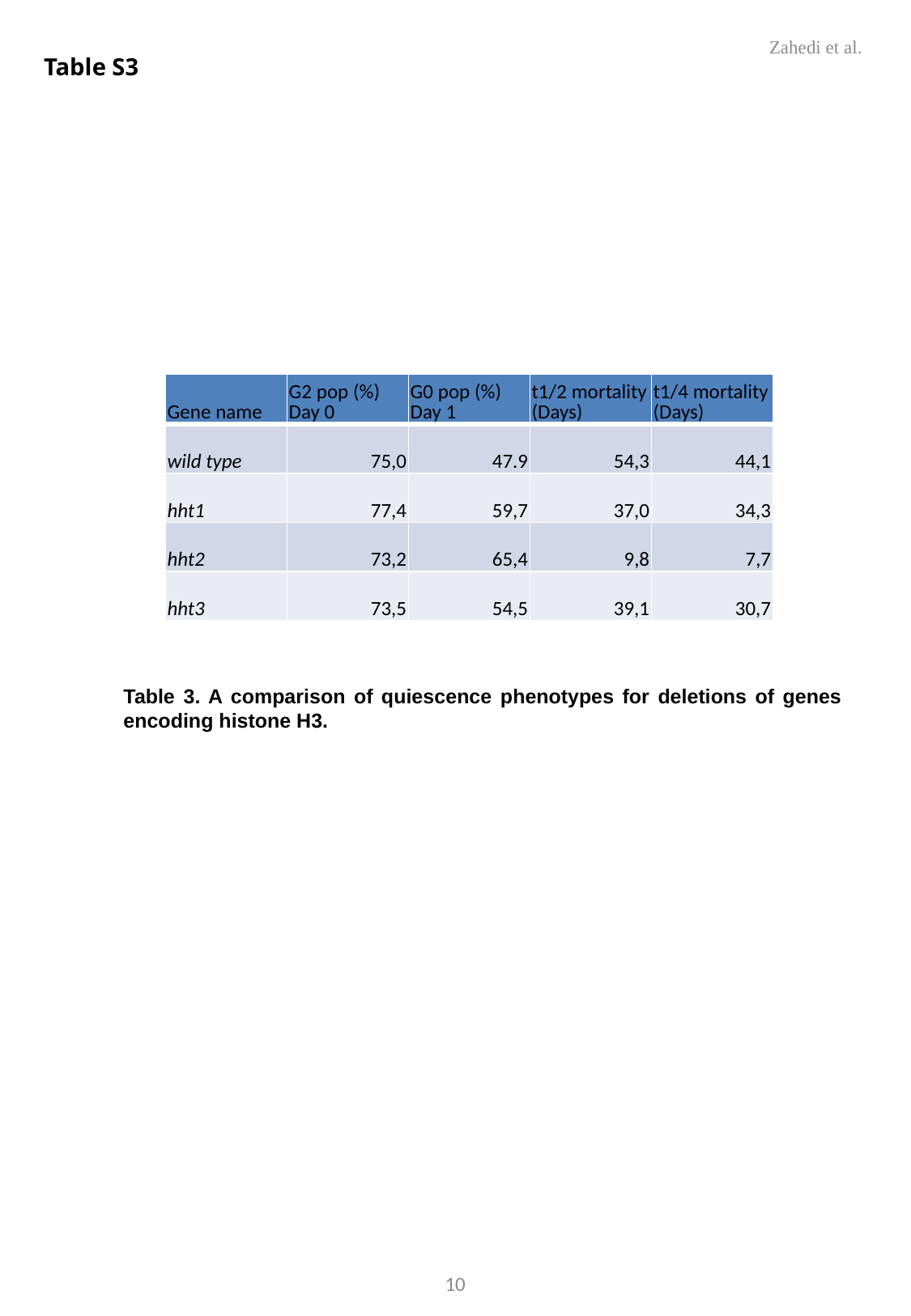

Zahedi et al.
Table S3
| Gene name | G2 pop (%) Day 0 | G0 pop (%) Day 1 | t1/2 mortality (Days) | t1/4 mortality (Days) |
| --- | --- | --- | --- | --- |
| wild type | 75,0 | 47.9 | 54,3 | 44,1 |
| hht1 | 77,4 | 59,7 | 37,0 | 34,3 |
| hht2 | 73,2 | 65,4 | 9,8 | 7,7 |
| hht3 | 73,5 | 54,5 | 39,1 | 30,7 |
Table 3. A comparison of quiescence phenotypes for deletions of genes encoding histone H3.
10
